# Prognostic significance of marital status in breast cancer survival: A population-based study

**DOI:** 10.1371/journal.pone.0175515

**Published:** 2017-05-05

**Authors:** María Elena Martínez, Jonathan T. Unkart, Li Tao, Candyce H. Kroenke, Richard Schwab, Ian Komenaka, Scarlett Lin Gomez

**Affiliations:** 1 Moores Cancer Center, University of California, San Diego, La Jolla, CA, United States of America; 2 Department of Family Medicine and Public Health, University of California, San Diego, La Jolla, CA, United States of America; 3 Department of Surgery, University of California, San Diego, La Jolla, CA, United States of America; 4 Cancer Prevention Institute of California, Fremont, CA, United States of America; 5 Kaiser Permanente, Division of Research, Oakland, CA, United States of America; 6 Maricopa Medical Center, Department of Surgery, Phoenix, AZ, United States of America; 7 Stanford Cancer Institute, Palo Alto, CA, United States of America; Sudbury Regional Hospital, CANADA

## Abstract

Research shows that married cancer patients have lower mortality than unmarried patients but few data exist for breast cancer. We assessed total mortality associated with marital status, with attention to differences by race/ethnicity, tumor subtype, and neighborhood socioeconomic status (nSES). We included, from the population-based California Cancer Registry, women ages 18 and older with invasive breast cancer diagnosed between 2005 and 2012 with follow-up through December 2013. We estimated mortality rate ratios (MRR) and 95% confidence intervals (CI) for total mortality by nSES, race/ethnicity, and tumor subtype. Among 145,564 breast cancer cases, 42.7% were unmarried at the time of diagnosis. In multivariable-adjusted models, the MRR (95% CI) for unmarried compared to married women was 1.28 (1.24–1.32) for total mortality. Significant interactions were observed by race/ethnicity (*P*<0.001), tumor subtype (*P*<0.001), and nSES (*P* = 0.009). Higher MRRs were observed for non-Hispanic whites and Asians/Pacific Islanders than for blacks or Hispanics, and for HR+/HER2+ tumors than other subtypes. Assessment of interactive effect between marital status and nSES showed that unmarried women living in low SES neighborhoods had a higher risk of dying compared with married women in high SES neighborhoods (MRR = 1.60; 95% CI: 1.53–1.67). Unmarried breast cancer patients have higher total mortality than married patients; the association varies by race/ethnicity, tumor subtype, and nSES. Unmarried status should be further evaluated as a breast cancer prognostic factor. Identification of underlying causes of the marital status associations is needed to design interventions that could improve survival for unmarried breast cancer patients.

## Introduction

A growing body of evidence shows that mortality following a cancer diagnosis is higher in unmarried than married patients [1–4]. However, studies on the association specific to breast cancer are limited [5, 6], despite this being the most common cancer in women. Some published reports included marital status as a covariate in multivariable analyses [7–9], but, to our knowledge, a focused examination of the specific effect of marital status on breast cancer survival in different racial/ethnic populations or by tumor subtype has not been reported.

A population-based study published in 2005 [6] showed that compared to married breast cancer patients, unmarried women were more likely to be diagnosed with later stage disease and to die of breast cancer, and were less likely to receive definitive treatment, even after controlling for stage, treatment, socioeconomic factors, and comorbidities. A more recent study conducted in a safety net hospital reported an over 2-fold increase in breast cancer mortality in single versus married breast cancer patients [5]. However, none of these studies examined whether the marital status effects on survival differ across patient subgroups or tumor subtypes.

We recently reported significant heterogeneity in the association between marital status and survival across racial/ethnic and nativity groups [4]; however, this research was not specific to breast cancer. Understanding the extent to which marital status impacts survival after breast cancer diagnosis across sociodemographic groups is needed to help target interventions. However, there are limited data regarding the extent to which marriage benefits survival in these groups. Importantly, since breast cancer is recognized to be a heterogeneous disease, data are needed on how the marital status associations with mortality differ by tumor subtype. Using data from the population-based California Cancer Registry (CCR), we conducted a comprehensive examination of the association of marital status and total mortality among women diagnosed with invasive breast cancer. Specifically, we assessed main effects of being unmarried and total mortality and whether the association varied by race/ethnicity, neighborhood socioeconomic status (nSES), and tumor subtype.

## Materials and methods

### Study population

We obtained from the CCR information on 152,380 female California residents age 18 and above who were diagnosed with a first primary invasive breast cancer [International Classification of Disease for Oncology, 3^rd^ Edition, (ICD-O-3) site codes C50.0–50.9, with the following ICD-O-3 coded histological subtypes of breast carcinoma or adenocarcinoma: 8000, 8001, 8010, 8020, 8022, 8050, 8140, 8201, 8211, 8230, 8255, 8260, 8401, 8453, 8480, 8481, 8500–8530, and 8575] during the period January 1, 2005 through December 31, 2012. We excluded patients who had no information on marital status at diagnosis (n = 6,179) and who were diagnosed by mammography/xerography (n = 70) or death certificate/autopsy (n = 567), and thereby included 145,564 patients in the final analysis.

CCR data on race, ethnicity, birthplace, and marital status were abstracted from medical records of reporting facilities and are based primarily on self-report. We also obtained from the CCR data on address at diagnosis (geocoded to census block group), American Joint Committee on Cancer (AJCC) stage, tumor size, lymph node involvement, grade, primary source of payment at the time of initial diagnosis and/or treatment, as well as treatment modalities (surgery, type of surgery, chemotherapy, and radiation therapy).

Birthplace is coded as US- or foreign-born. As previous research shows that birthplace is differentially missing in the cancer registry data between US- and foreign-born Hispanics and Asians/Pacific Islanders (APIs) [10, 11], we imputed nativity using patients’ social security numbers (SSN) for the 33% of Hispanics and 30% of APIs with missing registry birthplace. This validated imputation method assigns a foreign birthplace to Hispanic patients who received their SSNs after the age of 24 and to APIs after the age of 20 [12, 13]. Patient residential address at diagnosis was geocoded and assigned to a census block group, then linked to nSES indices developed through principal components analyses. The two indices, based on Census 2000 data (for cases diagnosed in 2005) and American Community Survey 2007–2011 data (for cases diagnosed 2006–2012), were developed separately but included the same components: education, occupation, employment, household income, poverty, rent and house values. [14, 15]. Vital status as of December 31, 2013 was obtained through linkages to various vital records databases.

The CCR has collected information on the expression of ER and PR since 1990 and of HER2 since 1999 [16]. Data completeness for HER2 is over 80% since 2005. Each marker was coded as positive, negative, borderline, not tested/recorded, or unknown, based on the results of the test performed at the reporting facility. We classified breast cancers into four mutually exclusive subtypes: HR+/HER2- was defined as ER and/or PR positive and HER2 negative; HR+/HER2+ as ER and/or PR positive and HER2 positive; HR-/HER2+ as ER and PR negative and HER2 positive; and triple-negative breast cancer as ER, PR, and HER2 negative [16–20]. The study was approved by the institutional review boards at each institution; informed consent was waived as we analyzed de-identified data.

### Statistical analysis

We used multivariable-adjusted Cox proportional hazards regression models to estimate hazard rate ratios (MRR) [21] and 95% confidence intervals (CI) [22] to evaluate differences in total mortality between married and unmarried (never married, separated, divorced, and widowed) breast cancer patients. The proportional hazards assumption was assessed for marital status and for each covariate by examining the correlation between weighted Schoenfeld residuals and logarithmically transformed survival time; no significant violations of the assumption were observed, except for stage. Thus, stage was included as a stratifying variable in all Cox regression models, allowing the baseline hazard to vary by stage. The models were adjusted for age, race/ethnicity, tumor subtype, treatment, lymph node involvement, tumor size, grade, histology, insurance status, and nSES. In our prior work, we found evidence of interaction between marital status and nSES; thus, statistical significance of multiplicative interaction effect of marital status and nSES was estimated with the Wald test by including a cross-product term in the Cox models. We also conducted separate analyses by race/ethnicity, tumor subtype, nSES, nativity among Hispanics and APIs, and for the six largest API ethnic groups: Chinese, Japanese, Filipino, Korean, South Asian, and Vietnamese. Cox regression and the Kaplan-Meier method was used to test statistical differences in overall survival by both marital status and nSES. All Cox regression models were adjusted for clustering by census block group. All statistical tests were carried out using SAS software version 9.3 (SAS Institute, Cary, North Carolina). All *P* values reported were two-sided, and those that were <0.05 were considered to be statistically significant.

## Results

### Characteristics of study population

Among the 145,564 breast cancer patients included in the analyses, 22,610 deaths from any cause occurred during a total of 620,692 person-years of follow-up. At the time of diagnosis, the percent of married, single/separated/divorced, and widowed women was 57.3%, 28.5%, and 14.2%, respectively. Table 1 shows that widowed women were more likely to be older and to be non-Hispanic white (NHW), and less likely to be uninsured than married or single patients. Married women were less likely to reside in low SES neighborhoods and more likely to have private insurance than unmarried women. In regard to tumor subtype, widowed patients had a lower proportion of HER2+ and triple negative tumors than other women. Widowed women were less likely to undergo surgery and to have chemotherapy or radiation therapy than other women.

**Table 1:** Sociodemographic, clinical, and treatment characteristics of patients diagnosed with breast cancer, according to marital status, California, 2005–2012.

	All N = 145564	Married N = 83383	Single/Separated/ Divorced N = 41521	Widowed N = 20660
	No. (%)	No. (%)	No. (%)	No. (%)
**Sociodemographic Characteristics**				
Age, years				
<35	3108 (2.1)	1665 (2.0)	1429 (3.4)	14 (0.1)
35–49	32321 (22.2)	21433 (25.7)	10512 (25.3)	376 (1.8)
50–59	37273 (25.6)	23943 (28.7)	11892 (28.6)	1438 (7.0)
60–64	18697 (12.8)	11408 (13.7)	5742 (13.8)	1547 (7.5)
65–69	16677 (11.5)	9531 (11.4)	4646 (11.2)	2500 (12.1)
70–74	12682 (8.7)	6677 (8.0)	2981 (7.2)	3024 (14.6)
≥75	24806 (17.0)	8726 (10.5)	4319 (10.4)	11761 (56.9)
Race/ethnicity				
Non-Hispanic white	90349 (62.1)	51715 (62.0)	24401 (58.8)	14233 (68.9)
Black	9324 (6.4)	3309 (4.0)	4627 (11.1)	1388 (6.7)
Hispanic	26103 (17.9)	15140 (18.2)	8108 (19.5)	2855 (13.8)
Asian/Pacific Islander	18374 (12.6)	12478 (15.0)	3896 (9.4)	2000 (9.7)
Other/unknown	1414 (1.0)	741 (0.9)	489 (1.2)	184 (0.9)
Neighborhood (block group) statewide SES quintile				
1st (lowest)	18450 (12.7)	8686 (10.4)	6815 (16.4)	2949 (14.3)
2nd	25289 (17.4)	13016 (15.6)	8106 (19.5)	4167 (20.2)
3rd	29720 (20.4)	16423 (19.7)	8814 (21.2)	4483 (21.7)
4th	34088 (23.4)	19988 (24.0)	9398 (22.6)	4702 (22.8)
5th (highest)	38017 (26.1)	25270 (30.3)	8388 (20.2)	4359 (21.1)
Insurance status				
No insurance	24509 (16.8)	14679 (17.6)	7476 (18.0)	2354 (11.4)
Private	67632 (46.5)	43916 (52.7)	18306 (44.1)	5410 (26.2)
Medicare or Medicare+Private	18742 (12.9)	9324 (11.2)	3715 (8.9)	5703 (27.6)
Any Public/Medicaid/Military	30452 (20.9)	13063 (15.7)	10907 (26.3)	6482 (31.4)
Unknown	4229 (2.9)	2401 (2.9)	1117 (2.7)	711 (3.4)
**Clinical Characteristics**				
AJCC Stage				
I	66046 (45.4)	39638 (47.5)	17082 (41.1)	9326 (45.1)
II	48760 (33.5)	28144 (33.8)	14194 (34.2)	6422 (31.1)
III	17754 (12.2)	9756 (11.7)	5711 (13.8)	2287 (11.1)
IV	6982 (4.8)	3084 (3.7)	2729 (6.6)	1169 (5.7)
Unknown	6022 (4.1)	2761 (3.3)	1805 (4.3)	1456 (7.0)
Molecular subtype				
HR+/HER2-	87989 (60.4)	50813 (60.9)	24510 (59.0)	12666 (61.3)
HR+/HER2+	14538 (10.0)	8573 (10.3)	4379 (10.5)	1586 (7.7)
HR-/HER2+	7274 (5.0)	4329 (5.2)	2116 (5.1)	829 (4.0)
Triple negative	15106 (10.4)	8705 (10.4)	4604 (11.1)	1797 (8.7)
Unclassified	20657 (14.2)	10963 (13.1)	5912 (14.2)	3782 (18.3)
Lymph node involvement				
Negative	92823 (63.8)	53962 (64.7)	25195 (60.7)	13666 (66.1)
Positive	48757 (33.5)	27961 (33.5)	14966 (36.0)	5830 (28.2)
Unknown	3984 (2.7)	1460 (1.8)	1360 (3.3)	1164 (5.6)
Tumor Size (cm)				
0.10 < tumor ≤0.50	9459 (6.5)	5863 (7.0)	2444 (5.9)	1152 (5.6)
0.50 <tumor ≤ 1.00	22449 (15.4)	13550 (16.3)	5620 (13.5)	3279 (15.9)
1.00 <tumor ≤ 2.00	48621 (33.4)	28997 (34.8)	13043 (31.4)	6581 (31.9)
2.00 <tumor ≤ 5.00	45579 (31.3)	25372 (30.4)	13711 (33.0)	6496 (31.4)
>5.00	11006 (7.6)	5474 (6.6)	3984 (9.6)	1548 (7.5)
Microinvasion	1546 (1.1)	962 (1.2)	414 (1.0)	170 (0.8)
Diffused	757 (0.5)	364 (0.4)	300 (0.7)	93 (0.5)
Unknown	6147 (4.2)	2801 (3.4)	2005 (4.8)	1341 (6.5)
Grade				
Grade I	30988 (21.3)	18109 (21.7)	8218 (19.8)	4661 (22.6)
Grade II	58240 (40.0)	33593 (40.3)	16211 (39.0)	8436 (40.8)
Grade III/IV	46587 (32.0)	26937 (32.3)	14123 (34.0)	5527 (26.8)
Unknown	9749 (6.7)	4744 (5.7)	2969 (7.2)	2036 (9.9)
Histology				
Ductal	111557 (76.6)	64600 (77.5)	32104 (77.3)	14853 (71.9)
Lobular	23418 (16.1)	13620 (16.3)	6262 (15.1)	3536 (17.1)
Other	10589 (7.3)	5163 (6.2)	3155 (7.6)	2271 (11.0)
**Treatment**				
Surgery				
No surgery	10368 (7.1)	4161 (5.0)	3783 (9.1)	2424 (11.7)
Breast conserving surgery	78579 (54.0)	46247 (55.5)	21609 (52.0)	10723 (51.9)
Mastectomy	56339 (38.7)	32845 (39.4)	16053 (38.7)	7441 (36.0)
Other/unknown	278 (0.2)	130 (0.2)	76 (0.2)	72 (0.3)
Chemotherapy				
No	83953 (57.7)	45097 (54.1)	22580 (54.4)	16276 (78.8)
Yes	59274 (40.7)	36972 (44.3)	18265 (44.0)	4037 (19.5)
Unknown	2337 (1.6)	1314 (1.6)	676 (1.6)	347 (1.7)
Radiation therapy				
No	76327 (52.4)	41365 (49.6)	22165 (53.4)	12797 (61.9)
Yes	69109 (47.5)	41978 (50.3)	19330 (46.6)	7801 (37.8)
Unknown	128 (0.1)	40 (0.0)	26 (0.1)	62 (0.3)

Abbreviations: SES = socioeconomic status; AJCC = American Joint Committee on Cancer; HR = hormone receptor; HER2 = human epidermal receptor 2

### Marital status and mortality

After multivariable adjustment, total mortality was 28% higher in unmarried compared to married patients (Table 2). Among unmarried patients, the MRR was significantly higher in widowed (MRR = 1.35; 95% CI, 1.30–1.41) than single/separated/divorced women (MRR = 1.24; 95% CI, 1.20–1.28), based on non-overlapping CIs.

**Table 2:** Adjusted mortality rate ratio (MRR) and 95% confidence intervals (CI) for total cancer mortality associated with marital status, California, 2005–2012.

	Total mortality	
	No. of deaths	MRR^a^ (95%CI)
**Marital status at diagnosis**	** **	** **
Married	9304	1.00 (Reference)
All unmarried	13306	1.28 (1.24–1.32)
Single/Separated/Divorced	6941	1.24 (1.20–1.28)
Widowed	6365	1.35 (1.30–1.41)

^a^Estimated from Cox proportional hazard models, stratified by stage (AJCC stage I-IV or unknown) and adjusted for: age (continuous); race/ethnicity (NHW, non-Hispanic white, black, Hispanic, Asian/Pacific Islander, other/unknown); tumor subtype HR+/HER2-, HR+/HER2+, HR-/HER2, triple negative, and unclassified); first course of treatment (Y/N for surgery, radiation, hormone therapy); lymph node involvement (negative, positive, unknown); tumor size (continuous); grade (I, II, II/IV, unknown); histology (ductal, lobular, other); insurance status (no insurance, private insurance only, Medicare only/Medicare + private insurance, any public/Medicaid/military insurance, unknown); and neighborhood socioeconomic status (quintiles).

We next assessed whether there was variation in the MRRs for marital status and mortality by race/ethnicity, nativity (among Hispanics and APIs), nSES, or tumor subtype (Table 3). Significant variation by race/ethnicity was shown (*P*-heterogeneity <0.0001), albeit the magnitude of the differences was modest. Among API ethnic groups, we noted significant associations for total mortality for Filipinos and other Asians, but numbers of events were limited in other ethnic groups. Assessing differences by nativity, no significant differences were shown among APIs, whereas significant variation was observed for Hispanics. The marital status-mortality associations were higher in US-born than foreign-born Hispanics (*P*-heterogeneity = 0.01) and significant variation by nSES was shown (*P*-heterogeneity = 0.009). Differences across tumor subtype for the marital status and mortality association were also evident, with higher MRRs observed for HR+/HER2- tumors (MRR = 1.32; 95% CI: 1.26–1.37) and lower for triple negative tumors (MRR = 1.16; 95% CI: 1.08–1.25). In all analyses performed, results for breast cancer-specific mortality were similar to those observed for all-cause mortality (data not shown). Also, since the proportion of individuals living with an unmarried partner in the U.S. has increased over time [23] and we are lacking these data in the registry, we restricted analyses to women <64 years of age, in whom cohabitation might be higher than in older patients. Results show no material differences in the MRRs as compared to the total population (data not shown).

**Table 3:** Adjusted mortality rate ratios (MRR) and 95% confidence intervals (CIs) for total mortality associated with unmarried vs. married patients, by race/ethnicity, tumor subtype, neighborhood socioeconomic status (SES), nativity, and Asian/Pacific Islander (API) subgroups, California, 2005–2012.

	Total mortality		
	No. of deaths Unmarried	No. of deaths Married	MRR^a^ (95%CI) Unmarried vs. Married
**By Race/ethnicity**	** **	** **	** **
Non-Hispanic white	8761	5858	1.31 (1.26–1.36)
Black	1599	614	1.17 (1.05–1.30)
Hispanic	1943	1750	1.15 (1.07–1.24)
Asian/Pacific Islander	894	1006	1.29 (1.16–1.43)
Other/unknown	109	76	1.06 (0.74–1.53)
*P*-heterogeneity^b^			<0.001
**Among APIs by ethnic group**			
Chinese	167	227	1.21 (0.95–1.54)
Japanese	125	92	1.33 (0.96–1.83)
Filipino	305	321	1.24 (1.03–1.50)
Other Asian	138	135	1.35 (1.02–1.79)
Korean	52	68	1.38 (0.89–2.15)
South Asian	47	84	0.84 (0.53–1.33)
Vietnamese	60	79	1.03 (0.68–1.56)
*P*-heterogeneity^b^			0.6183
**Among Hispanics by nativity**			
US-born	948	727	1.23 (1.10–1.37)
Foreign-born	995	1023	1.07 (0.97–1.18)
*P*-heterogeneity ^b^			0.010
**Among APIs by nativity**			
US-born	168	128	1.62 (1.25–2.11)
Foreign-born	722	876	1.25 (1.12–1.41)
*P*-heterogeneity^b^			0.425
**By Neighborhood SES**			
1st (lowest)	2526	1357	1.22 (1.13–1.32)
2nd	2918	1804	1.26 (1.18–1.35)
3rd	2857	2009	1.33 (1.24–1.42)
4th	2751	2068	1.22 (1.14–1.30)
5th (highest)	2254	2066	1.32 (1.24–1.42)
*P*-heterogeneity ^b^			0.009
**By Tumor Subtype**			
HR+/HER2-	5939	4102	1.32 (1.26–1.37)
HR+/HER2+	1157	899	1.18 (1.07–1.30)
HR-/HER2+	779	705	1.21 (1.07–1.35)
Triple negative	1961	1803	1.16 (1.08–1.25)
Unclassified	3470	1795	1.35 (1.25–1.46)
*P*-heterogeneity^b^			<0.0001

^a^Estimated from Cox proportional hazard models, stratified by stage (AJCC stage I-IV or unknown) and adjusted for: age continuous); race/ethnicity (NHW, non-Hispanic white, black, Hispanic, Asian/Pacific Islander, other/unknown); tumor subtype HR+/HER2-, HR+/HER2+, HR-/HER2, triple negative, and unclassified); first course of treatment (Y/N for surgery, radiation, hormone therapy); lymph node involvement (negative, positive, unknown); tumor size (continuous); grade (I, II, II/IV, unknown); histology (ductal, lobular, other); insurance status (no insurance, private insurance only, Medicare only/Medicare + private insurance, any public/Medicaid/military insurance, unknown); and neighborhood socioeconomic status (quintiles).

^b^Likelihood ratio test for interaction computed based on cross-product terms.

**Fig 1:**
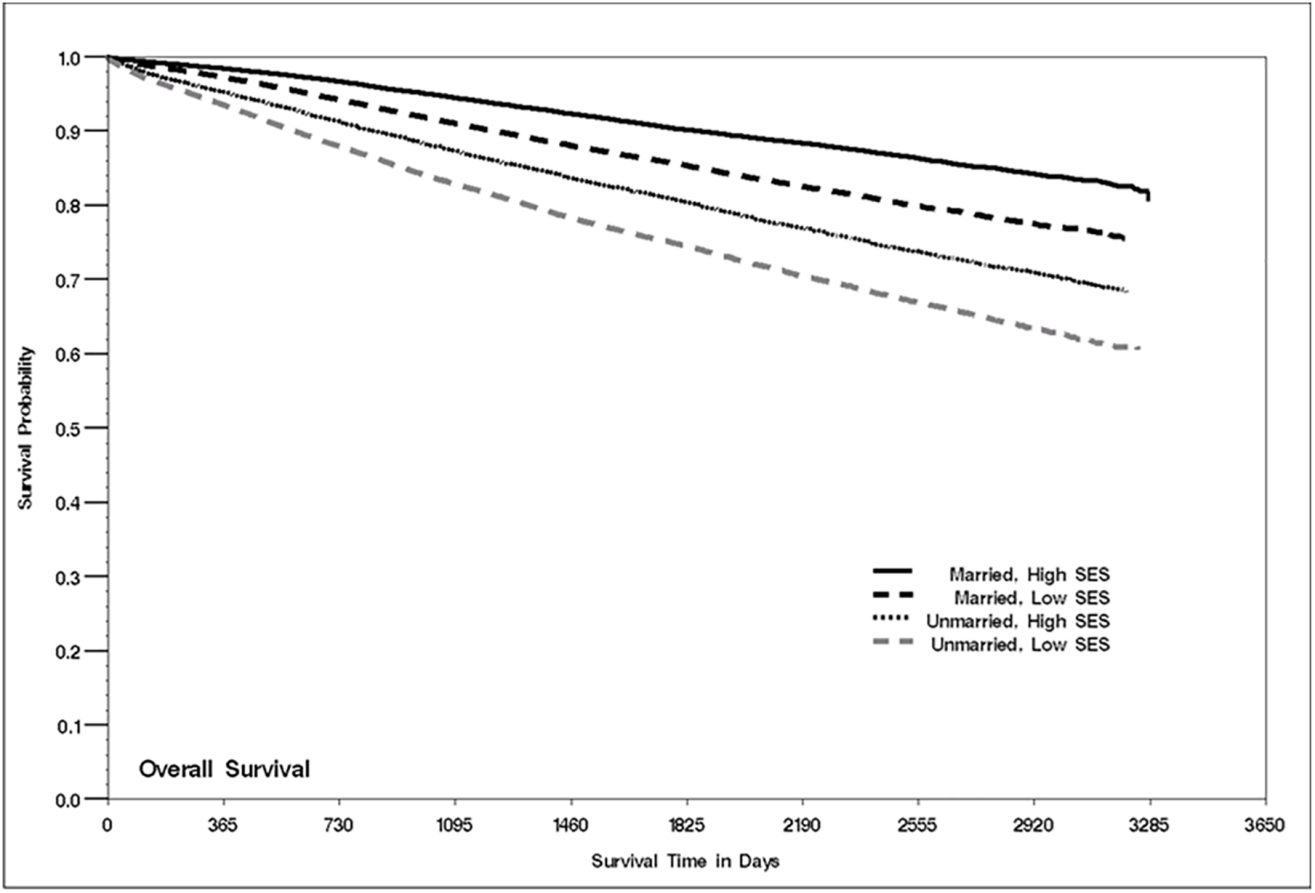
Kaplan-Meier curve of all-cause survival by marital status and neighborhood SES, California, 2005–2012.

**Fig 2:**
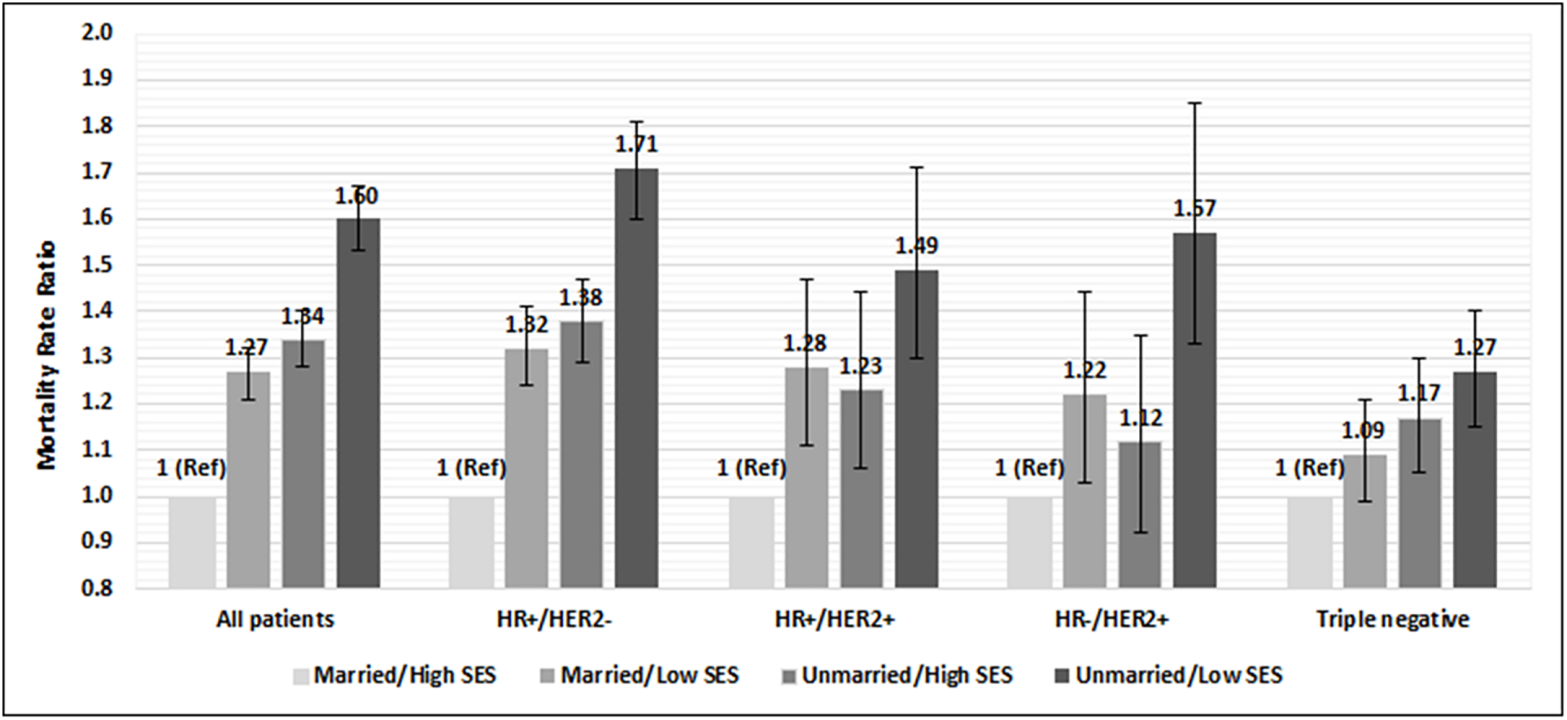
Hazard ratios for total mortality and marital status plus neighborhood socioeconomic status (SES), California, 2005–2012. Hazard ratios estimated from Cox proportional hazard models adjusted for: age at diagnosis (continuous), race/ethnicity, subtype (for analysis of all patients), lymph node involvement, tumor size, grade, histological subtypes, first course of treatment, insurance status; AJCC stage I-IV or unknown is included as a stratifying variable. High SES group includes neighborhood SES quintiles 4–5 and low SES group includes quintiles 1–3. Numbers represent hazard ratio and bars represent 95% confidence interval for each group. *Denotes statistical significance, whereby the confidence intervals do not include 1. Abbreviations: MRR = hormone receptor; HER2 = human epidermal receptor 2; Ref = reference group.

Lastly, we assessed mortality and the interactive effects of marital status and nSES. As shown in Fig 1, survival was highest among married women who resided in high SES neighborhoods and lowest among unmarried women in low SES neighborhoods. When we conducted multivariable analyses, a similar pattern emerged (Fig 2 and S1 Table). Compared to married women who resided in high SES neighborhoods, patients with all other combinations of marital status (married/unmarried) and SES (low/high) had higher risk of total mortality. In all women, the strongest association was shown for unmarried patients living in low SES neighborhoods (MRR = 1.60; 95% CI, 1.53–1.67), which was also evident when we stratified by tumor subtype. The largest mortality difference by SES/marital status group was seen for women with HR+/HER2- and the smallest was observed for patients with triple negative tumors (Fig 2).

## Discussion

Results of our population-based study show that breast cancer patients who were unmarried at diagnosis had a significantly higher risk of dying of all causes compared with those who were married. Additionally, larger associations among NHW and API women, US-born Hispanics and APIs, and patients diagnosed with HR+/HER2- tumors were observed.

Although there is a growing body of evidence on the adverse effects of being unmarried compared to being married on cancer mortality [1, 3, 4, 24–26], data specific to breast cancer as well as the effect of marital status on mortality by race/ethnicity are sparse, as prior studies have lacked sufficient sample size to evaluate these associations. Our prior research showed higher mortality rates among foreign-born relative to US-born APIs with breast cancer, but lower mortality among foreign-born relative to US-born Hispanics [27, 28]. The smaller effects for marital status on mortality among foreign-born API patients and lack of marriage effect among foreign-born Hispanics in the current study may contribute to the understanding of factors underlying the survival differences between immigrants and their US-born counterparts, which merits further study. Specifically, API immigrant breast cancer patients may face more extreme burdens, such as language barriers, cultural beliefs, and immigration-associated stress, that limit their ability to benefit from quality access to care and receipt of guideline treatments; these ethnic- and culturally-specific factors may diminish the potentially beneficial effects of marriage. On the other hand, immigrant Hispanic breast cancer patients may have already strong social ties and support from their co-ethnic social networks, that any additional benefits of marriage may be minimal [29].

We know of no published reports where the association of marital status and mortality in breast cancer patients has been assessed according to tumor subtype. Given that breast cancer is considered a heterogeneous disease, this is an important consideration. Indeed, results of our study showed significant heterogeneity by tumor subtype, with highest association observed in HR+/HER2- and lowest in triple negative tumors. Triple negative breast cancer is known to be an aggressive subtype with poor prognosis and few treatment options [16]. The beneficial effects of marriage seen in other tumor subtypes with longer life expectancy may not be as relevant in aggressive and rapidly-progressing tumors characterized by few treatment options. This is consistent with our prior report [3], where MRRs for total mortality and marital status were stronger in cancers with better prognosis, including prostate and non-Hodgkin lymphoma.

Published data on the positive associations between being married and undergoing mammography screening [30] and with breast cancer treatment uptake [31, 32] may explain some of the associations between marital status and mortality following breast cancer diagnosis, although the beneficial effect of marriage on breast cancer survival persists after accounting for treatment [6]. Two main pathways have been proposed to explain the benefits of marital status on cancer and overall longevity [33]: better economic resources and greater social support. In regard to the first pathway, we previously assessed the effect of nSES and health insurance status on overall survival from the 10 most common cancers [3]. Our results suggested that the higher mortality associated with being unmarried vs. married was not explained by the availability of financial resources [34]. MRRs unadjusted for nSES and health insurance were 1.27 for males and 1.19 for females and attenuated slightly to 1.22 and 1.15, respectively, after adjustment for these two economic-related variables. In fact, the significant interaction seen in the analysis of the cross-classification of marital status and nSES in this report suggests that the lack of neighborhood resources may compound, rather than explain, the effect of unmarried status on outcomes. Marital ties increase social network size not just through availability of a partner but through access to the partner’s network ties, while nSES may reflect social capital as well as proxy of individual-level SES. Significant additive and interactive effects seen in these analyses suggest that both high SES neighborhoods and marital ties each confer different and critical resources predictive of survival.

Relative to the social support pathway, prior research shows better breast cancer survival among patients with a larger number of social ties [2, 35–37] and with greater social support [38, 39]. However, data on these specific contextual factors and whether they mediate or moderate the marital status association are not known. Women’s social relationships have been shown to influence choice of mastectomy or lumpectomy [40], whether to pursue chemotherapy [41], and other treatment decisions [41]. A marital partner may also provide critical support that helps improve health behaviors. In a recent study, women with breast cancer who were unmarried were more likely to be current smokers and less likely to receive chemotherapy [42]. Cluze et al. [43] also reported that a larger number of family members or friends supporting the breast cancer patient was associated with better adherence to adjuvant endocrine therapy. Clinical implications of these and our current results are evident, including awareness among oncologists and other cancer providers to recognize unmarried patients as a high-risk group for higher mortality. Consequently, it may be beneficial to involve nurse case managers, clinical social workers and/or psychologists at various points during a patient’s prolonged treatment course. For the research community, it is important to note that data on social factors and social support as an explanatory factor for the reported association between marital status and mortality in cancer patients are limited, requiring much needed work focused on exploring possible mechanisms to guide future interventions. Biologic mechanisms potentially responsible for poor social support and isolation associated with breast cancer progression include immune, endocrine function, and stress-related factors [44], variables that have been linked with tumor growth and progression in breast cancer animal models and human studies [45–47].

Results of our study must be interpreted in light of the limitations. Although it could be argued that assessing marital status at time of diagnosis is appropriate in terms of timing, we could not assess changes in marital status following a breast cancer diagnosis. It is also important to note that we lacked information on co-habitation, support from children and other family members, and quality of marriage. Cancer registry-recorded race, ethnicity, and birthplace may be subject to some misclassification; however, because this information is usually based on self-report (extracted from patient medical records) [48], it is generally accurate for most racial/ethnic groups [10, 11, 49–51]. However, because registry birthplace data are incomplete in a biased manner, we used a validated approach to impute nativity. Further, we lacked information on comorbidities, specific treatment modalities, which could be potential mediators or confounders in our analyses. Specifically, we do not have data on psychological and cultural factors, or levels of social support. As a result, we are not able to address specific contextual factors responsible for the observed associations between marital status and mortality. Our results may not be generalizable beyond the California population. We cannot dismiss the possibility of self-selection, whereby women who are physically, emotionally, or psychologically healthier may be more likely to marry than those who are not. In addition, selection out of marriage due to divorce might also contribute to this self-selection.

Our results show that breast cancer patients who are unmarried have higher all-cause mortality compared to married patients, but that this survival benefit varies across racial/ethnic groups, by tumor subtype, and nSES. These findings along with the growing body of evidence on this topic underscore the importance of identifying and examining the reasons for the gap in mortality in married versus unmarried patients. These data are urgently needed so that we can identify and implement targeted interventions that could help ameliorate the poorer survival among unmarried breast cancer patients.

## Supporting information

S1 Table Adjusted hazard ratios (MRR) and 95% confidence intervals (CIs) for total mortality associated with marital status plus neighborhood socioeconomic status (SES), California, 2005–2012. Abbreviations: HR = hormone receptor; HER2 = human epidermal receptor 2; Q = quintile.
^a^Estimated from Cox proportional hazard models adjusted for: age at diagnosis (continuous), race/ethnicity, subtype (for analysis of all patients), lymph node involvement, tumor size, grade, histological subtypes, first course of treatment, insurance status; AJCC stage I-IV or unknown is included as a stratifying variable.
(DOCX)
