# Supplementary material for: Prognostic significance of marital status in breast cancer survival: A population-based study
Source: PLoS One. 2017 May 5;12(5):e0175515. doi: 10.1371/journal.pone.0175515 (PMC5419505; doi:10.1371/journal.pone.0175515)
Supplement: S1 Table — Abbreviations: HR = hormone receptor; HER2 = human epidermal receptor 2; Q = quintile. aEstimated from Cox proportional hazard models adjusted for: age at diagnosis (continuous), race/ethnicity, subtype (for analysis of all patients), lymph node involvement, tumor size, grade, histological subtypes, first course of treatment, insurance status; AJCC stage I-IV or unknown is included as a stratifying variable. (DOCX) [file pone.0175515.s001.docx]

**S1 Table**. **Adjusted hazard ratios (MRR) and 95% confidence intervals (CIs) for total mortality associated with marital status plus neighborhood socioeconomic status (SES), California, 2005-2012**

|  | **Total mortality** | |
| --- | --- | --- |
|  | **No. of deaths** | **MRR^a^ (95%CI)** |
| **All** |  |  |
| Married, High SES (Q4-5) | 4134 | 1.00 (Reference) |
| Married, Low SES (Q1-3) | 5170 | 1.27 (1.21-1.32) |
| Unmarried, High SES (Q4-5) | 5005 | 1.34 (1.28-1.40) |
| Unmarried, Low SES (Q1-3) | 8301 | 1.60 (1.53-1.67) |
| **HR+/HER2-** |  |  |
| Married, High SES (Q4-5) | 1919 | 1.00 (Reference) |
| Married, Low SES (Q1-3) | 2183 | 1.32 (1.24-1.41) |
| Unmarried, High SES (Q4-5) | 2381 | 1.38 (1.29-1.47) |
| Unmarried, Low SES (Q1-3) | 3558 | 1.71 (1.60-1.81) |
| **HR+/HER2+** |  |  |
| Married, High SES (Q4-5) | 392 | 1.00 (Reference) |
| Married, Low SES (Q1-3) | 507 | 1.28 (1.11-1.47) |
| Unmarried, High SES (Q4-5) | 392 | 1.23 (1.06-1.44) |
| Unmarried, Low SES (Q1-3) | 765 | 1.49 (1.30-1.71) |
| **HR-/HER2+** |  |  |
| Married, High SES (Q4-5) | 290 | 1.00 (Reference) |
| Married, Low SES (Q1-3) | 415 | 1.22 (1.03-1.44) |
| Unmarried, High SES (Q4-5) | 258 | 1.12 (0.92-1.35) |
| Unmarried, Low SES (Q1-3) | 521 | 1.57 (1.33-1.85) |
| **Triple negative** |  |  |
| Married, High SES (Q4-5) | 810 | 1.00 (Reference) |
| Married, Low SES (Q1-3) | 993 | 1.09 (0.99-1.21) |
| Unmarried, High SES (Q4-5) | 716 | 1.17 (1.05-1.30) |
| Unmarried, Low SES (Q1-3) | 1245 | 1.27 (1.15-1.40) |
| **Unclassified** |  |  |
| Married, High SES (Q4-5) | 723 | 1.00 (Reference) |
| Married, Low SES (Q1-3) | 1072 | 1.30 (1.16-1.46) |
| Unmarried, High SES (Q4-5) | 1258 | 1.53 (1.36-1.72) |
| Unmarried, Low SES (Q1-3) | 2212 | 1.63 (1.47-1.82) |

Abbreviations: HR=hormone receptor; HER2=human epidermal receptor 2; Q=quintile.

^a^Estimated from Cox proportional hazard models adjusted for: age at diagnosis (continuous), race/ethnicity, subtype (for analysis of all patients), lymph node involvement, tumor size, grade, histological subtypes, first course of treatment, insurance status; AJCC stage I-IV or unknown is included as a stratifying variable.
